# Specific aspects of tympanoplasty in children: A retrospective cohort study of 95 cases

**DOI:** 10.1016/j.amsu.2021.102297

**Published:** 2021-04-19

**Authors:** Sanaa Mallouk, El Bouhmadi Khadi, Walid Bijou, Youssef Oukessou, Rouadi Sami, Larbi Abada Redallah, Mahtar Mohammed

**Affiliations:** ENT Department, Face and Neck Surgery, Hospital August, 20’1953, University Hospital Center IBN ROCHD, Hassan II University, Casablanca, Morocco

**Keywords:** Tympanoplasty, Children, Chronic otitis media, Success rate

## Abstract

**Background:**

Paediatric tympanoplasty is now a common surgical procedure. The age from which it could be proposed varies regarding children specificities such as Eustachian tube dysfunction, the high incidence of upper airway infections and the immaturity of the immune system. The aim of this study is to describe the specific constitutional, epidemiological and operative aspects as well as the anatomical and functional results of tympanoplasty in children.

**Methods:**

From 2014 to 2018, a cohort of 95 patients with ages between 6 and 16 years, operated for a type I tympanoplasty, was reviewed by analysing the medical history, the epidemiological and clinical parameters, in addition to the operative features and the functional results.

**Results:**

The mean age at surgery was 11,7 years. The main risk factors of tympanic perforation were recurrent otitis (78,9%), auricular trauma (16,8%) and tonsillar and adenoid hypertrophy (7,4%). Good anatomical postoperative results with a closed and reinforced neo-tympanic membrane were seen in 90 (94,7%) cases, while a significant improvement of the hearing loss was observed in 87 (91.6%) patients, with a mean value of 34,23 dB HL before and 21,9 dB HL after surgery (p < 0,0001).

**Conclusions:**

The indications of type I tympanoplasty in the paediatric population remain a subject of debate, but still offer good anatomical and functional results as long as it is adapted to each particular case.

## Highlights

o The indications of type I tympanoplasty in children remain a subject of debate.
o Eustachian tube maturity influences the result.
o Treatment of chronic otitis media prevents tympanic perforations.
o Good outcome is obtained if surgery is adapted to each child.

## 1 Introduction

Type I tympanoplasty or myringoplasty is a common surgical procedure involving reconstruction of a perforated tympanic membrane, restoring its anatomical and functional integrity with no intervention on the ossicular chain [1], using fascia temporalis, conchal and/or tragal cartilage grafts and/or perichondral grafts [2].

In the paediatric population, the high incidence of upper airways infections leads to chronic otitis media and tympanic perforations. Thus, tympanoplasty became a usual intervention, with a reported prognosis different from that described in adults.

The main specific aspects of the disease in children are the peculiar narrowness of the external auditory canal, the frequent dysfunction of Eustachian tube and the presence of adenoid hypertrophy. However, tympanoplasty in children remains a matter of debate concerning the optimal age to perform the surgery and the factors thought to influence surgical outcome [3].

The aim of the study was to describe the specific epidemiological and surgical findings as well as the anatomical and functional outcomes of tympanoplasty in children in light of the existing literature.

## 2 Patients and methods

A retrospective cohort study was carried out over a 5-years period, between January 2014 and December 2018, in the Otorhinolaryngology and Head and Neck department of the August 20, 1953 hospital. The clinical, functional and operative data were collected based on the analysis of 136 files of which only the 95 cases with comprehensive data were retained. The inclusive criteria were the age between 6 and 16 years, with a dry perforation resulting from chronic suppurative otitis media with no ossicular erosion.

The otoscopic examination was based on 0° oto-endoscope then under microscope. The perforations were classified as “small” if concerning less than 25% of the tympanic membrane, “medium” between 25 and 50%, and “large” over 50%; while their situation was either anterior, posterior, central or total. The determination of the size of perforation was made pre-operatively. We started by delimitating the 4 tympanic quadrants, identified between the vertical line parallel to the malleus manubrium and the second line perpendicular to it going through the malleus umbo. Then we evaluated the perforation according to the concerned quadrants.

The hearing evaluation was based on bilateral pure tone audiometry, calculating the mean hearing loss and the air-bone gap for both ears.

The surgical procedure was performed on dry ears. Patients with discharging ears were then treated preoperatively by topical Fluoroquinolones for duration of 10 days.

The retro-auricular approach was adopted for all the patients, under microscope, through type I underlay tympanoplasty technique, using the fascia temporalis, the conchal cartilage or the perichondral graft. The ossicular chain was evaluated per-operatively. Routine prophylactic antibiotic therapy was given to all patients for a week.

The follow up was scheduled at day 21, then 3 months, 6 months and a year after surgery. Anatomical success was defined as the presence of an intact graft evaluated by 0°oto-endoscope without perforation, atelectasis nor lateralization at least 6 months post-operatively.

Functional result was assessed by comparing the pre and post-operative air-bone gap (0,5 to 4 kHz) according to the criteria of the Committee on hearing and equilibrium for the evaluation of results of treatment of conductive hearing loss [4].

The statistical analysis evaluating hearing improvement (comparison of mean hearing loss and mean air-bone gap) was based on the khi-2 test of Pearson, considering the correlation significant with a p < 0,05.

The work has been reported in line with the STROCSS criteria [4], approved by the ethical committee of our department and is registered under this identifying number: researchregistry6617.

## 3 Results

Among the 95 collected patients, there were 58 males (61,06%) and 37 females (38,94%), the male/female ratio was 1,56:1. The age of surgery ranged from 6 to 16 year-old with a median age of 11,7 years. Of all patients, 75 (78,9%) of them had a history of recurrent otitis, 16 (16,8%) had a history of auricular trauma. 9 (9,5%) had a previous tympanoplasty, 7 (7,4%) had an adenoidectomy and tonsillectomy and 1 (1,1%) had a tympanostomy tube placement.

The most common presenting clinical manifestation was persistent otorrhea in 88 cases (92,6%), followed by intermittent otorrhea in 13 cases (13.7%) and hearing loss in 13 cases (13.7%). Only 5 patients presented with otalgia (5.3%).

The clinical examination found a unilateral perforation in 89 of the cases (left in 51,57% and right in 42,11%) and bilateral in 6 patients. The antero-inferior site was the most frequent (32,64%) followed by the postero-inferior and subtotal (20%) then the central and total (2,1%) (Table 1). Also, the contralateral ear was normal in 87 patients (82,67%), perforated in 6 patients (6,32%), with a tympanic retraction pocket in 1 case (1,05%) and already operated for a type I tympanoplasty 2 years ago in 1 patient (1,05%).

**Table 1:** Patterns of tympanic membrane perforation.

Location of perforation		N (%)
Anterior	Antero-inferior	31 [6,32]
	Antero-superior	11 [7,11]
Posterior	Postero-inferior	19 [20]
	Postero-superior	13 [7,11]
Subtotal		19 [20]
Central		1 [1]
Total		1 [1]

The pre-operative PTA revealed unilateral conductive hearing loss (CHL) in 75 patients (78,94%), unilateral mixed hearing loss (MHL) in 2 cases (2,11%) and bilateral conductive hearing loss in 4 patients (4,21%).

All the perforated ears were clean and dry before surgery, topical antibiotics were used in case of infection. The procedure was carried out under general anaesthesia. Surgical exploration found an intact ossicular chain. The middle ear mucosa was inflammatory in 54 cases (56,8%) and normal in the other 41 cases. The graft for the tympanic reconstruction was applied by an underlay technique. The fascia temporalis was used in 51 patients (53,68%), reinforced by conchal cartilage in 28 patients (29,47%) or by tragal cartilage in 11 patients (11,59%).

The immediate post-operative was uneventful in the high majority of cases, with the occurrence of a fever in 7,4% of the cases, vertigo in 2,1%, vomiting in 1,1% and otorrhea in 1,1%. The late follow up on a period of at least 12 months showed good results with a closed and reinforced neo-tympanic membrane in 94,7% of the cases.

However, for the last 5 patients, the anatomical success as defined previously was compromised. Retraction pocket appeared 18 months later in 1 patient whom reconstruction material was a fascia temporalis graft. 4 patients (4,2%) had a reperforation occurring in a median of 14,75 months postoperatively. Their initial perforations were subtotal in 50% of the cases and the grafts used were fascia temporalis and conchal grafts in 3 patients and tragal cartilage graft alone in one patient (Table 2).

**Table 2:** Types of failed anatomical results.

Type of complication	Site of initial perforation	Reconstruction material	Time to occurrence (months)
Reperforation 1	Posterior	Temporalis fascia + Conchal cartilage	24
Reperforation 2	Subtotal	Temporalis fascia + Conchal cartilage	12
Reperforation 3	Subtotal	Temporalis fascia + Conchal cartilage	12
Reperforation 4	Postero-inferior	Tragal cartilage	11
Retraction pocket	Postero-inferior	Temporalis fascia	18

The functional results were evaluated by a pure tone audiometry. The mean hearing loss was up to 21,9 dB (15–50 dB) with a mean air-bone gap at 9,96 dB (10–40 dB). The comparison with the preoperative findings showed a significant improvement of the hearing loss after tympanoplasty, 34,23 dB before and 21,9 dB after surgery, with p < 0,0001. Also, the mean ear-bone gap decreased significantly from 21,6 dB to 9,96 dB, with p < 0,0001. More specifically, from the 95 operated patients, hearing improvement was seen in 87 and was <10 dB in 61.1% of the cases, between 11 and 30 dB in 30,5% of the cases and absent in 8,4% of the cases (Table 3).

**Table 3:** Post-operative gain results.

Mean gain (dB HL)	N (%)
0	8 (8.4%
<10	58 [1,61]
10–30	29 [5,30]

## 4 Discussion

Tympanic membrane perforation in children is a well-known sequel of chronic and recurrent otitis media leading to multiple infections and hearing loss with an eventual impact on language and learning [3]. Thus, even if its parameters are controversial, tympanoplasty became indispensable.

However, children are still considered as bad candidates for surgery regarding the Eustachian tube immaturity and frequent dysfunction as well as the high incidence of upper airways infections [5]. Thereby, the ideal age of surgery varies and the French society of Otorhinolaryngology (SFORL) recommends the detection of underlying sinonasal affection and the treatment of all the recurrent upper air ways infections before tympanoplasty [6]. Indeed, Hamans et al. [7] recommend the treatment of adenoid vegetations first as well as for Charachon et al. [8], while the tonsillectomy is indicated only in front of chronic tonsillitis or obstructive tonsils. Contrariwise, Pignataro et al. [9] report no benefit of adenoid vegetations cure before tympanoplasty. Also, an observation delay seems necessary since the rate of spontaneous closing of the tympanic membrane is considerable, in a limit of 6 months [10]. All in all, the success rate of paediatric tympanoplasty is variable, evaluated at 35–94% versus 60–99% in adults [11].

The gender didn't seem to influence the results in our study as well as on Kaya et al.‘s study [11] who reported a sex ratio of 1:1 and stated that it is not related to the postoperative result.

Clinically, 93,68% of our patients presented unilateral perforations majorly in the left side while bilateral perforations were seen in 6,32%. Castro et al. [12] reported also a predominance of left unilateral perforations. While the literature considers the site of the perforation as the only significant anatomical predictive factor of surgical success [1], Ern Tan et al. [13] found this significant impact more on the size of the perforation, with a lower rate of success for perforations of more than 50% of the tympanic membrane. And Al-Khtoum et al. [14] reported a higher incidence of surgical failure in cases of total perforation.

The state of the contralateral ear is important to evaluate. Eustachian tube dysfunction can lead to a reperforation by middle ear aeration impairment. Poor eustachian-tube function has been considered as an explanation by some authors as to why younger age may be correlated with lower tympanoplasty success rates [15]. Also, contralateral hearing loss imposes a softer surgical technique with minimal manipulation of the ossicular chain [16].

Hearing assessment found a mean air-bone gap value pf 21,6 dB HL in our study, 27,4 dB HL in Kaya et al.‘s [11]. serie and 28,75 dB HL in Al Khtoum et al. [14] serie. Also, the degree of hearing loss, up to 34,23 dB in our study, can vary according to the underlying etiology. Chronic otitis media usually generate a conductive hearing loss up 50 dB in some cases [17], while post traumatic perforations have a better functional prognosis, causing only mild hearing loss, rarely exceeding 30 dB [18].

The indications of tympanoplasty are variable and depend on anatomical and clinical criteria. For Koch et al. [19], children with a pars tensa perforation persistent after 6 months should be treated surgically. Prescott et al. [20] report that a persistent otorrhea despite adequate medical treatment is a surgical indication. The perforations secondary to tympanostomy tubes persistent after 6 months to 1 year should also be treated surgically [16]. However, perforations secondary to burn injury never heal spontaneously [21].

Performing tympanoplasty on ears with active drainage after drying the ear is recommended by many authors [[22], [23], [24]]. The surgery can be postponed up to 12 months in cases of wet ear [25]. since as in Pignatora et al. [9], the success rate was higher for tympanoplasty in dry ears thanks to a better integration of the graft. However, the *French Society of Otolaryngology* (SFORL) also proposes surgery on a wet ear with active and persistent otorrhea despite medical treatment and management of risk factors [16]. Indeed, Caylan et al. study [26] reports a better tympanic healing with a rate of 100% on a wet ear versus 75% on a dry one, since good and proliferating tympanic membrane vascularisation favours a better and quicker cicatrisation of the graft.

In the paediatric population, tympanoplasty is performed under general anaesthesia [16] through different approaches. The retroauricular approach adopted in our serie, and by the majority of authors [23,27,28], offers good exposition of the donor site and an optimal access of the middle ear especially in case of anterior perforation. The latter can be difficult to visualize because of the convexity of the anterior wall of the auditory canal in children. The main drawbacks to this approach are a larger scar and the fair amount of soft tissue dissection [28].

In comparison, a higher rate of failure was seen with the transcanal approach without tympanomeatal flap [23]. Halim et al. [29], when comparing 102 paediatric tympanoplasties, didn't observe any significant difference between the endaural and retroauricular approach.

Multiple biomaterials or autologous grafts can be used for the tympanic reconstruction. The fascia temporalis is characterized by its accessibility and its reliability [30]. The conchal and tragal cartilages offer mechanical stabilization and resistance with a mild loss on the acoustic transfer. Some authors report a better morphological result on the negative pressure effect of the middle ear that can lead to retraction pockets [31]. Perichondral grafts, used with cartilage, undergo only little modifications and assure good resistance on Eustachian tubes dysfunction [16,32].

The “underlay” technique is widely used as in our study and consists of placing the graft entirely medial to the remaining drum and malleus. In contrast, the overlay technique is more challenging and typically reserved for total perforations, anterior perforations, or failed underlay surgery; and consists of placing the graft lateral to the annulus and any remaining fibrous middle layer after the squamous layer has to be carefully removed [33].

The endoscopic tympanoplasty has the advantage of a high resolution vision of the hardly exposed regions as the hypotympanum, the sinus tympani, the posterior wall of the mesotympanum and the epitympanum [34], however, the placement of the graft can be constrained by the endoscope in children [16].

The postoperative follow up in the paediatric population varies among studies. For Uyar et al. [35] the mean follow up period was up to 63,6 months (12–143 months), while it extended on 9 months for Harkani et al. [10] and 12 months in our study.

The main complications after tympanoplasty are reperforation, retraction, graft lateralization, anterior blunting iatrogenic cholesteatoma [16]. Early reperforation (<3 months following surgery) are related to the surgical technique (non-adequate placement of the graft, insufficient recovery of the graft by the canal epithelium), postoperative infection or a hyperpressure secondary to blowing [36]. Delayed reperforation (>3 months following surgery) testifies of underlying middle ear pathology. Use of fascia temporalis graft alone, regarding its flexibility, can favour retraction pocket, particularly in children [36] The incidence of iatrogenic cholesteatoma, especially with the overlay technique can reach 4.4% [35].

Success of pediatric tympanoplasty is a controversial topic. The exhaustive definition of tympanoplasty success on children includes the complete cicatrisation and integrity of the graft within the tympanic membrane in an anatomical position, without atelectasis or middle ear otitis or effusion, with a minimal auditory gain of 10 dB or a preservation of hearing levels [37]. The disparity of outcomes in pediatric tympanoplasty is related to heterogenity of the patients included, different definition of success and heterogenity of the patients included and the post-operative follow-up period. Age, size and location of perforation, status of the operated and contralateral ear, presence of hypertrophic adenoids, function of the auditory tube and surgeons’ experience are factors that are independent of surgical success [3]. Isaacson et al. [28] series showed a success rate of 61%. On another hand, Uyar et al. [35] study on 41 child reported 19,4% of anatomical failures with 12,2% of graft lateralization, 4,8% of reperforation and 2,4% of retraction pockets. Also, Anatomical failure was up to 20% out of 60 tympanoplasties in Harkani et al. serie [10]. In this study the success rate was 94,7%. It is a relatively high rate according to data found on the literature. This could be explained by the age of the patients included in the study and the duration of the follow up a period.

To sum up, paediatric tympanoplasty is a common surgical intervention where the age from which it could be proposed varies regarding children specificities such as Eustachian tube dysfunction, the high incidence of upper airway infections and the immaturity of the immune system [29]. The absence of these factors in adults explains the higher success rate, up to 60–99% versus 35–94% in children [38]. The local state of the ear is a parameter discussed in both populations. The surgical approach depends on the used graft, the site and size of the perforation and the surgeon preferences. According to Ern Tan et al. [13], the surgical approach does not influence the result while the cartilage graft showed superiority upon other types of grafts.

The limitation of this study is mainly the follow-up duration since a longer follow-up is warranted to come with definitive conclusions.

## 5 Conclusion

The indications of type I tympanoplasty in children remain a controversial question and should be adapted to each case in order to obtain the highest rate of success. However, the efficient prevention of chronic otitis media stills the most adequate tool to prevent tympanic perforations secondary to chronic inflammation and recurrent infection.

## Ethical approval

This work has been approved by the ethical committee of our department.

## Funding

None.

## Registration of research studies

Researchregistry6617.

### Informed consent

Informed consent was obtained from all individual participants included in the study.

## Provenance and peer review

Not commissioned, externally peer-reviewed.

## Author contribution

Sanaa MALLOUK: Corresponding author, Writing the paper.

Khadija ELBOUHMADI : Writing the paper.

Walid BIJOU : Correcting the paper.

Youssef OUKESSOU : Study concept, Writing the paper.

Samid ROUADI : Study concept.

Reda ABADA : Correcting the paper.

Mohamed ROUBAL : Correcting the paper.

Mohamed MAHTAR : Correcting the paper.

## Guarantor

 Sanaa MALLOUK.

## Declaration of competing interest

The authors declare that they have no conflicts of interest concerning this article.
